# Material-Driven Clinical Complications in Mechanical Circulatory Support: From Blood–Material Interactions to Device-Related Adverse Events

**DOI:** 10.3390/ma19122683

**Published:** 2026-06-22

**Authors:** Klaudia Cholewa, Agnieszka Szuber-Dynia, Jakub Włodarczyk, Klaudia Kurtyka, Artur Kapis, Sachiro Kakinoki, Przemysław Kurtyka, Roman Major, Maciej Gawlikowski

**Affiliations:** 1 Faculty of Biomedical Engineering, Silesian University of Technology, 41-800 Zabrze, Poland; klaudia.cholewa@polsl.pl (K.C.); agnieszka.szuber-dynia@polsl.pl (A.S.-D.); maciej.gawlikowski@polsl.pl (M.G.); 2 Institute of Heart Prostheses, Foundation of Cardiac Surgery Development, 41-800 Zabrze, Poland; akapis@frk.pl; 3 Centre of Polymer and Carbon Materials, Polish Academy of Sciences, Cmpw PAN, 41-800 Zabrze, Poland; jwlodarczyk@cmpw-pan.pl (J.W.); kkurtyka@cmpw-pan.pl (K.K.); 4 Department of Chemistry and Materials Engineering, Faculty of Chemistry, Materials and Bioengineering, Kansai University, Osaka 564-8680, Japan; sachiro@kansai-u.ac.jp; 5 Institute of Metallurgy and Materials Science, Polish Academy of Sciences, 30-059 Kraków, Poland; r.major@imim.pl

**Keywords:** mechanical circulatory support, extracorporeal pulsatile systems, clinical complications, biocompatibility, local infections, thrombogenicity, prosthetic valves

## Abstract

Mechanical circulatory support (MCS) has transformed the management of advanced heart failure; however, device-related morbidity remains substantially driven by adverse interactions occurring at the blood–material and tissue–device interfaces. Despite progressive miniaturization and the evolution from first-generation pulsatile systems to contemporary continuous-flow devices, thrombotic, hemorrhagic, infectious, and inflammatory complications continue to limit long-term outcomes. This review examines the mechanistic contribution of material properties, surface architecture, and hemodynamic conditions to the pathogenesis of major MCS-associated complications, with particular emphasis on thrombogenicity, biomaterial-induced inflammatory activation, driveline and cannulation-associated infections, hemocompatibility disturbances, and device-related structural failure. The interplay between protein adsorption, platelet activation, complement cascade dysregulation, disturbed shear profiles, and biofilm formation is analyzed as a central determinant of adverse clinical events. Special attention is given to pediatric MCS, in which the continued reliance on extracorporeal pulsatile systems, unique anatomical constraints, and narrow therapeutic margins intensify susceptibility to both thromboembolic and infectious sequelae. Furthermore, the review addresses how material and surface modifications, and emerging biomimetic and anti-thrombogenic coatings may influence complication mitigation. By integrating clinical, engineering, and biomaterials perspectives, this work highlights that many complications traditionally regarded as secondary clinical phenomena are fundamentally rooted in device–material interactions and flow-mediated biological responses. Improved understanding of these mechanisms is essential for optimizing device design, enhancing hemocompatibility, and reducing complication burden in both adult and pediatric MCS populations.

## 1. Introduction

### 1.1. The Burden of Heart Failure as a Determinant of MCS Utilization

The increasing demand for mechanical circulatory support (MCS) reflects the growing global burden of heart failure, which remains one of the leading causes of morbidity and healthcare utilization worldwide. Contemporary estimates based on the Global Burden of Disease (GBD) 2021 framework indicate that heart failure affected approximately 55.5 million individuals globally in 2021, with an age-standardized prevalence of approximately 676 per 100,000 population [1]. The scale of this problem is also consistently supported by independent clinical and epidemiological sources. According to major cardiovascular society reports, heart failure affects approximately 1–2% of the adult population in developed countries, with prevalence exceeding 10% among individuals over 70 years of age [2,3]. These estimates are derived from population cohorts, registry data, and healthcare system analyses, and are consistent with GBD-based observations.

Long-term epidemiological studies indicate that the burden of heart failure is driven not only by increasing incidence but also by improved survival among patients with cardiovascular disease. Advances in acute cardiac care have resulted in a growing population of patients living with chronic and progressive myocardial dysfunction, many of whom eventually progress to advanced stages of heart failure [4]. This shift has been described as a transition from acute cardiovascular mortality to chronic cardiovascular morbidity, with heart failure representing a common final pathway of multiple cardiac conditions [4]. Importantly, the epidemiology of heart failure is no longer limited to elderly populations. Although age remains the dominant risk factor, an increasing prevalence in younger adults has been observed, reflecting the contribution of cardiomyopathies, congenital heart disease, and metabolic disorders [4]. This trend has direct implications for MCS, as younger patients are more likely to be considered for advanced therapies. Pediatric heart failure represents a distinct subgroup with specific etiological and clinical characteristics. Although less prevalent than adult heart failure, it constitutes a clinically significant population, primarily driven by congenital heart disease and cardiomyopathies, which directly influence the need for temporary and paracorporeal mechanical circulatory support [5,6].

In Poland, the burden of heart failure is best interpreted through healthcare system data that directly inform resource allocation and planning of advanced therapies. Approximately 1.2 million individuals were living with heart failure in Poland in 2021, corresponding to about 3.2% of the general population [7]. Long-term analyses indicate a substantial increase in disease burden in recent years, with the number of patients rising by approximately 34% between 2014 and 2019 and reaching 1.02 million cases [7].

Registry-based analyses further confirm that heart failure in Poland is characterized not only by high prevalence but also by exceptionally high healthcare utilization. A nationwide observational study based on Ministry of Health and Health Needs Map data demonstrated that more than one million patients were treated for heart failure between 2014 and 2021, with over 9.2 million healthcare services recorded in this population. Notably, hospitalization rates remain among the highest across OECD countries, reaching 616 per 100,000 population in 2019, nearly three times the international average [8]. Furthermore, the number of hospitalizations exceeded the number of treated patients by 18–25%, indicating a high rate of recurrent admissions and reflecting the chronic, decompensating nature of the disease [8]. The burden of heart failure in Poland is not limited to clinical outcomes but also extends substantially into the economic domain. Analyses of indirect costs have shown that heart failure imposes a considerable societal and public finance burden, with total indirect costs increasing from €871.9 million in 2012 to €945.3 million in 2015, corresponding to approximately 0.21–0.22% of the national GDP. Mortality-related productivity losses represented the dominant component of these costs, accounting for more than 60% of the total indirect burden [9]. These findings indicate that heart failure is not only a clinical problem but also a major socioeconomic challenge affecting workforce participation and healthcare expenditure. Recent data extending into 2024 and early 2025 indicate a continued increase in the number of patients entering the healthcare system with a diagnosis of heart failure, further reinforcing the upward trend observed over the past decade [10]. These administrative and payer-based datasets capture the population actively receiving care and therefore more accurately reflect the real demand placed on healthcare infrastructure, including advanced heart failure therapies. The Polish epidemiological profile is characterized by high prevalence, exceptionally high hospitalization rates, significant mortality, and substantial economic burden. These features define a healthcare environment in which demand for advanced therapies, including MCS, is expected to increase. At the same time, high rates of recurrent hospitalization and prolonged disease trajectories imply extended exposure to device-related complications, making the interaction between epidemiology and device performance particularly relevant in this setting.

From the perspective of MCS, epidemiological data define not only the size of the potential candidate population but also the scale of exposure to device-related complications. As the number of patients with advanced heart failure increases and the duration of support extends, the absolute burden of complications such as infection, thrombosis, embolic events, and reintervention grows proportionally [1,4]. The expanding population of patients progressing to end-stage heart failure, cardiogenic decompensation, and transplant candidacy translates directly into a growing cohort exposed to prolonged MCS. Complication rates are not determined solely by clinical management but are increasingly influenced by duration of support and device-related factors. This relationship establishes a direct link between epidemiology and device performance. The rising number of patients requiring MCS amplifies the clinical impact of limitations at the blood–device and tissue–device interfaces, including material-dependent processes such as thrombogenicity and local exit-site infection. As a result, even stable individual complication rates may translate into a substantial increase in the overall clinical burden at the healthcare-system scale.

### 1.2. Pediatric Emphasis on First Generation MCS Systems

MCS systems are commonly classified into three generations according to pump design and device configuration. First-generation systems are pulsatile, paracorporeal devices in which blood flow is generated by the movement of a flexible membrane. In most systems, membrane displacement is driven by compressed air delivered by an external pneumatic driving unit, which alternates the filling and ejection phases of the blood chamber, while prosthetic valves ensure unidirectional flow. Second-generation devices are continuous-flow axial pumps, whereas third-generation systems are centrifugal pumps incorporating magnetic or hydrodynamic levitation. These design features were introduced to reduce shear stress and thrombogenicity associated with earlier continuous-flow devices, particularly those related to high impeller speeds and mechanical bearings. This technological progression reflects a shift from external pulsatile ventricular assist devices (VADs) toward smaller implantable rotary blood pumps; however, it has not eliminated the clinical relevance of first-generation MCS systems in children [11,12]. Their continued use reflects persistent limitations related to chest and heart size, vascular access, and anatomical variability, particularly in infants and small children [13]. Size-related constraints highlight an unresolved mismatch between pediatric anatomy and the physical dimensions, inflow requirements, and implantation constraints of rotary pumps developed primarily for adult circulation [14,15]. Contemporary clinical reports consistently indicate that paracorporeal pulsatile VADs, most commonly the Berlin Heart EXCOR, remain the primary option for long-term support in the pediatric population [16]. Data from contemporary pediatric registries, including the Pedimacs report published in 2024, indicate that MCS in children is used primarily as a bridge strategy rather than as destination therapy [17]. At 6 months after implantation, favorable outcomes, defined as transplantation, recovery, or ongoing support, are achieved in approximately 84–85% of patients. Heart transplantation remains a major endpoint, although recovery is also observed in selected patient groups, particularly those with potentially reversible myocardial dysfunction [17].

## 2. Material-Related Mechanisms of Local Infection in MCS

The implantation of first-generation paracorporeal pulsatile MCS devices introduces structural features that are directly relevant to complication risk [18]. For left ventricular support, the inflow cannula is typically placed in the left atrium or ventricular apex, whereas the outflow cannula is connected to the ascending aorta. Both cannulae are surgically attached to the cardiovascular system and pass through the skin, where they are externally connected to the pump chamber. In standard clinical practice, the cannulae are commonly externalized through the upper abdominal quadrant, which provides a relatively stable and accessible site for fixation, wound care, and dressing management.

**Figure 1:**
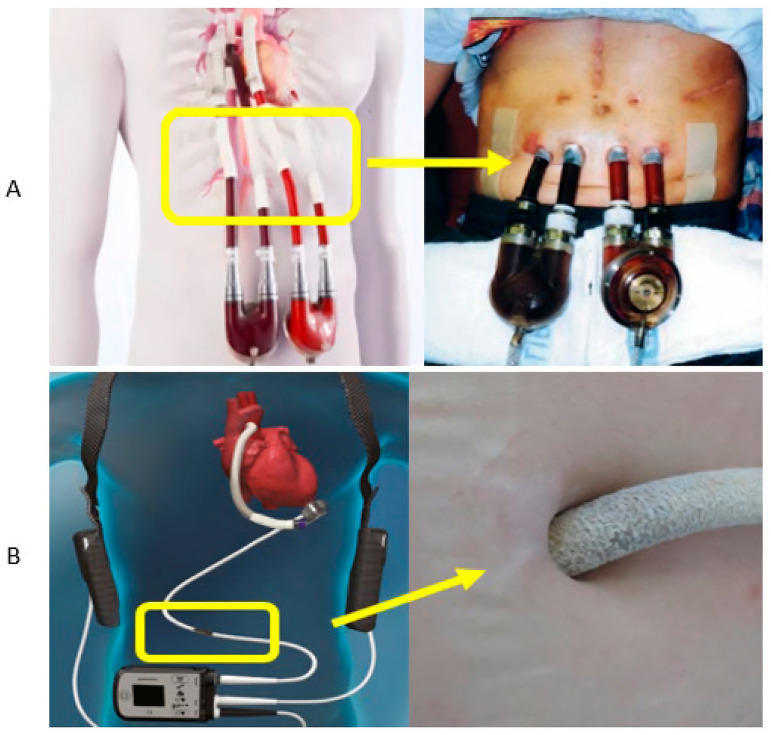
Schematic and clinical images of percutaneous access sites: (**A**) cannulas of a first-generation extracorporeal pulsatile pump (Thoratec Paracorporeal BiVAD, Thoratec Corporation, Pleasanton, CA, USA); (**B**) driveline of an implantable continuous-flow rotary pump (HeartMate III, Abbott Medical, Pleasanton, CA, USA) [18,19,20,21,22]. The yellow markings on the schematic images indicate the percutaneous access sites and their corresponding appearance in the clinical images.

As a result, at least two permanent percutaneous exit sites areprepared for the inflow and outflow cannulae. In patients requiring biventricular support, the number of cannulae and exit sites increases to four. Figure 1 presents a schematic representation of percutaneous access sites in MCS system [18].

Each exit site represents a permanent interruption of skin integrity and requires regular local care, including dressing changes and secure fixation of the cannula. Compared with implantable continuous-flow systems, which typically involve a single driveline, paracorporeal pulsatile devices require larger cannula exit sites and therefore create broader areas of contact between the external device components and surrounding soft tissues [15,16,18].

Interpretation of local infections in MCS requires standardized terminology. The International Society for Heart and Lung Transplantation (ISHLT) introduced criteria for the classification of infections in patients supported with ventricular assist devices, which are now widely used in clinical studies and registries [18,23,24]. These definitions allow more consistent distinction between infection type, anatomical extent, and severity, improving the comparability of outcomes reported by different centers.

From the perspective of material-related complications, standardized definitions are important because they determine which processes occurring at the contact zone between the device and surrounding tissue are reported as device-related infections. Local infections in MCS are most frequently observed where device components pass through the skin, including driveline exit sites in implantable systems and cannula exit sites in paracorporeal devices. These sites are characterized by loss of skin integrity and are exposed to mechanical stress, repeated local care, and the external environment. One of the major contributing factors is micro-motion at the exit site. Repeated movement of the driveline or cannula may cause local tissue injury, delay wound healing, and facilitate bacterial entry. Clinical observations indicate that instability at the driveline exit site is associated with an increased risk of infection [25]. In addition, the presence of a polymeric, porous material creates a local environment with limited immune response and reduced antibiotic penetration, which promotes persistence of microorganisms after initial colonization [26]. Biofilm formation on material surfaces represents another important mechanism. Polymeric materials used in percutaneous components of MCS systems, including Dacron (PET), polytetrafluoroethylene (PTFE), and silicone, facilitate bacterial adhesion and biofilm development. Early-stage biofilm formation is dominated by initial bacterial adhesion to the material surface and is strongly influenced by surface chemistry, wettability, roughness, and the conditioning layer of adsorbed host proteins. At this stage, bacterial attachment remains relatively reversible and microorganisms are more susceptible to antimicrobial therapy and host immune response. Mature biofilm is characterized by the formation of a complex bacterial structure, which limits antimicrobial penetration, reduces immune clearance, and promotes persistent bacterial survival, thereby favoring chronic or recurrent infection. In the case of silicone, additional material-related aspects should also be considered. Experimental and clinical studies of extracorporeal blood circuits have demonstrated that silicone components subjected to repetitive mechanical stress may undergo particle shedding, referred to as spallation, leading to the release of material fragments into the bloodstream and their subsequent deposition in tissues. Although these observations originate primarily from dialysis-related systems rather than long-term MCS cannulae, they indicate that silicone used in blood-contacting components may be susceptible to mechanical degradation under chronic loading conditions [27,28]. This observation is relevant for the evaluation of long-term polymer stability in mechanically active blood-contacting systems. Biofilms are less susceptible to antimicrobial therapy and more resistant to host immune response, which is associated clinically with recurrent or persistent exit-site infections and progression to deeper or systemic infections [18,23]. These mechanisms indicate that local infection is not determined solely by microbial exposure or patient-related factors, but is also influenced by material properties, surface characteristics, and mechanical conditions at the interface between tissue and device. In pediatric MCS, first-generation pulsatile systems introduce additional risk due to the presence of larger percutaneous cannula exit sites than the driveline one.

Clinical data support the relevance of these mechanisms. Registry analyses, including Pedimacs data, show that infection remains one of the most common adverse events in children supported with MCS, particularly during prolonged support [17]. The presence of multiple exit sites, combined with repeated handling during routine care, increases opportunities for contamination and microbial colonization. In adult populations, infection also remains a major complication despite advances in pump technology. Clinical trial and registry data indicate that infections continue to contribute substantially to morbidity and mortality. In the MOMENTUM 3 trial, infection was among the most frequently reported adverse events, with no significant reduction observed despite improvements in pump design [29]. Similarly, INTERMACS data show that infection and sepsis remain among the leading causes of death in patients supported with MCS [30]. These findings are particularly relevant in the context of technological progress: while modern pump design has reduced thrombosis and hemolysis, infection has remained a persistent complication, underscoring the importance of the local interaction between device components and surrounding tissue.

Local infections also remain a major limitation of implantable MCS systems. Their incidence is influenced by both mechanical and clinical factors. Mechanical properties such as driveline or cannula diameter, stiffness, and flexibility affect motion at the exit site and the extent of local tissue trauma. Clinical observations suggest that lower driveline stiffness and greater flexibility may reduce infection risk by limiting micro-motion and mechanical irritation of surrounding tissues [25,26,29]. In addition, repeated handling of the exit site, including dressing changes, is associated with increased infection risk because of local tissue disturbance and repeated exposure to external contaminants [23]. The persistence of infection as a major complication of MCS indicates that improvements in hemocompatibility alone are insufficient to reduce the overall complication burden. Increasing attention is therefore directed toward the material surface and its contact with surrounding tissue. Current research focuses on biomaterials with improved antibacterial properties and surface modifications designed to reduce bacterial adhesion and biofilm formation. These approaches include passive strategies that limit protein and bacterial adsorption, as well as active surface modifications incorporating antimicrobial or bioactive agents, including zinc oxide, silver, copper, titanium dioxide, antibiotic-eluting systems, smart dressings, and antibacterial peptides [18]. Metal-based antibacterial systems remain among the most extensively investigated approaches; however, their translation to long-term blood-contacting or tissue-contacting components requires careful control of antimicrobial activity, cytocompatibility, coating stability, and release kinetics. Silver-containing materials are active against a broad spectrum of microorganisms, but uncontrolled ion release, potential cytotoxicity, and loss of long-term activity remain important limitations. Zinc oxide and copper-based systems also show antibacterial potential; however, their stability, local tissue response, and suitability for chronic implantation require further evaluation before they can be considered appropriate for MCS-related applications [18].

Another strategy supported by broader biomaterials research is low-temperature plasma-assisted surface activation. This approach allows selective modification of the outermost material layer without altering the bulk properties of the polymer, which is particularly important for flexible percutaneous components exposed to long-term mechanical loading. Depending on process parameters, plasma treatment may introduce polar functional groups, increase surface energy, and modify wettability, thereby affecting protein adsorption and the early stages of bacterial adhesion. Plasma activation may also be used as a preparatory step for subsequent surface functionalization with antimicrobial or anti-adhesive chemistries, while preserving the mechanical performance of the underlying material [31,32]. For MCS components, low-temperature plasma appears to be the most suitable plasma-based approach, as polymeric blood-contacting materials may undergo thermal deformation, degradation, or changes in mechanical properties under high-temperature conditions.

Taken together, these observations indicate that prevention of MCS-related local infections requires strategies directed not only against microbial contamination, but also against the material and mechanical conditions that support bacterial persistence. For percutaneous components, the main direction is the development of multifunctional surfaces that combine reduced bacterial adhesion, controlled antimicrobial activity, mechanical durability, and acceptable tissue response. This approach is particularly relevant for cannula and driveline exit sites, where the material remains in long-term contact with tissue and is repeatedly exposed to mechanical stress, dressing changes, and the hospital environment. Future biomaterial development should therefore integrate surface chemistry, surface topography, polymer stability, and local tissue compatibility, rather than rely on a single antimicrobial additive. This direction is especially relevant for pediatric paracorporeal pulsatile systems, in which larger cannula exit sites and prolonged support increase the clinical significance of material-related infection risk.

## 3. Valve-Related Limitations in First-Generation Pulsatile MCS Systems

The material-related mechanisms discussed above are not limited to percutaneous components. In first-generation pulsatile systems, blood is also repeatedly exposed to internal polymeric surfaces, cannula connectors, membranes, and valves during each filling and ejection cycle. Although these elements are not involved in exit-site infection in the same manner as percutaneous components, their material properties and geometry strongly influence local flow conditions. Regions of recirculation, stagnation, or elevated shear stress may promote protein adsorption, platelet activation, and thrombus formation.

One important and often underrepresented limitation of first-generation pulsatile MCS systems concerns the valves used within the pump. Although paracorporeal pulsatile devices remain clinically important, particularly in pediatric patients because they do not require intrathoracic implantation and allow direct visual assessment of pump function through transparent housing, their design continues to rely largely on prosthetic heart valves originally developed for intracardiac implantation [30,33]. In current pulsatile MCS systems and total artificial hearts, these valves are adapted from surgical valve replacement applications rather than designed specifically for operation within extracorporeal blood pumps. This introduces several technological and clinical limitations.

**Figure 2:**
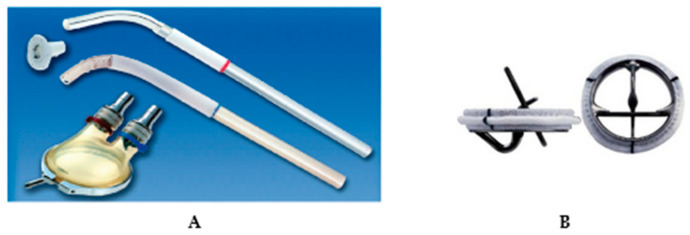
(**A**) Pulsatile paracorporeal MCS pump Toyobo [36], (**B**) Valves utilized in Toyobo: Medtronic Hall [37].

A major challenge is dependence on the commercial availability of specific valve models. Discontinuation or limited availability of these products requires manufacturers to modify pump construction and repeat validation procedures, which are time-consuming and costly. Historical and contemporary systems illustrate this issue. The Toyobo extracorporeal MCS system, introduced clinically in Japan in 1982, also used mechanical heart valves, including Medtronic Hall valves (Figure 2) [34,35].

**Figure 3:**
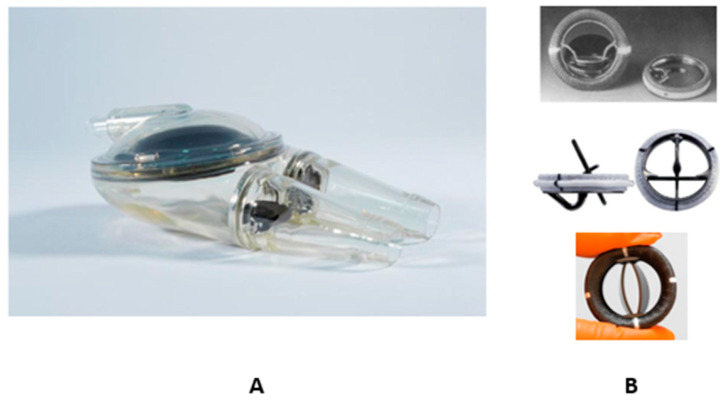
(**A**) Pulsatile paracorporeal MCS pump POLVAD-MEV [39], (**B**) Valves used in the POLVAD-MEV system: previously utilized Sorin Carbocast [40] and Medtronic Hall [37] valves, and the currently used bileaflet CorCym Bicarbon Fitline valve [self-developed data].

A similar approach was adopted in the Thoratec PVAD, which incorporated tilting-disc mechanical valves and was used extensively in clinical practice, with approximately 5000 patients treated [38]. Dependence on commercially available valve components remains evident in more recent systems. In the Polish POLVAD-MEV device, the valve models used in the pump have been modified over time according to market availability, from Sorin Carbocast and Medtronic Hall valves to the currently used CorCym Bicarbon Fitline valve [37,39,40,41]—Figure 3.

**Figure 4:**
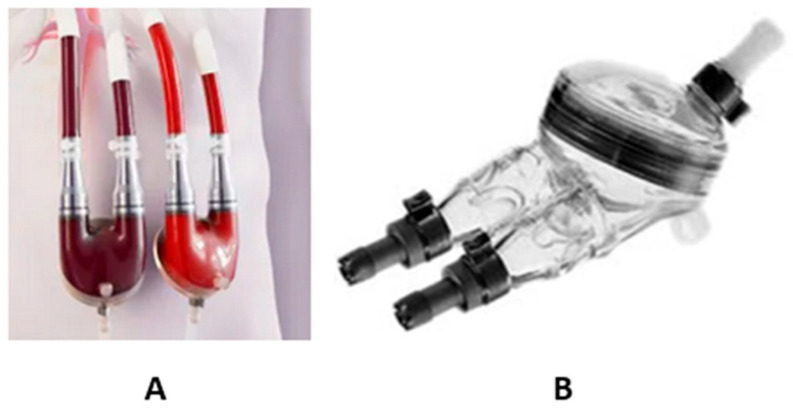
(**A**) Pulsatile paracorporeal MCS pump Berlin Heart EXCOR with mechanical valves [20]; (**B**) Pulsatile paracorporeal MCS pump Berlin Heart EXCOR with polyurethane trileaflet valves [44].

In Berlin Heart EXCOR devices, both mechanical valves and polyurethane trileaflet valves are used depending on pump configuration [20,42,43,44]—Figure 4.

**Figure 5:**
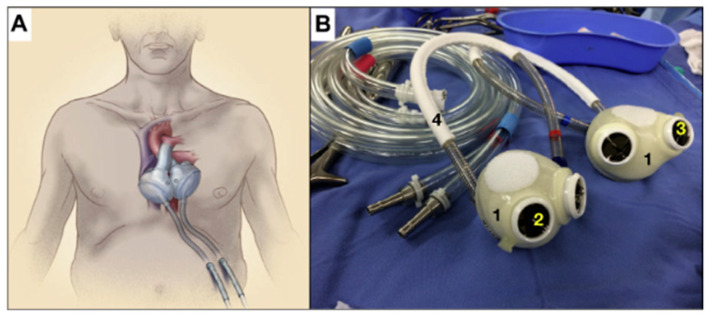
(**A**) SynCardia total artificial heart after implantation, (**B**) SynCardia device before implantation: 1—blood chamber, 2—inflow mechanical valve, 3—outflow mechanical valve, 4—pneumatic driveline connecting the prosthesis to the external driver [46].

Similarly, the SynCardia total artificial heart incorporates valves derived from implantable designs, including the mechanical single-disc SynHall valve [45]—Figure 5.

In addition to supply-related constraints, the structural design of many mechanical valves introduces important flow-related limitations. Mechanical valves contain support elements that retain and guide the valve disc or leaflets, and these structures are located directly within the blood flow path. Their presence may disturb local hemodynamics and contribute to flow separation, recirculation, stagnation, and locally increased shear stress. These disturbances can impair washout of the pump chamber and create conditions favorable for thrombus formation. This is particularly relevant in pulsatile systems, where cyclic filling and ejection increase sensitivity to geometric irregularities within the flow path. Cost considerations also contribute to the limitations of current solutions. Mechanical prosthetic valves are relatively expensive components, and their use directly increases the overall cost of pulsatile support systems, which may further restrict their availability and widespread application. These observations indicate that the use of heart valves prostheses not specifically designed for mechanical circulatory support remains a significant limitation of first-generation pulsatile systems. Dependence on external suppliers, variability in product availability, and geometric constraints affecting flow all contribute to technological and clinical challenges. For this reason, the development of valve designs dedicated to extracorporeal blood pumps represents an important direction for further advancement of MCS systems. Polyurethane valves are of particular interest, as their geometry can be adapted to the shape of the pump blood chamber. This allows for improved flow conditions, a reduction in stagnation zones, and potentially lower risk of thrombus formation. In addition, polymer-based valve designs may facilitate the application of antithrombogenic surface coatings on blood-contacting elements. Such approaches may be particularly beneficial during the early period after device implantation, when systemic anticoagulation is often limited and the hemocompatibility of the pump surface plays a key role in reducing initiation of coagulation and early thrombus formation.

Polyurethane heart valves have attracted considerable interest due to their favorable mechanical properties and the possibility of tailoring their geometry to specific flow conditions within blood pumps. However, despite these advantages, their long-term durability remains a significant concern. One of the main limitations associated with polyurethane valves is their tendency toward calcification, which can lead to structural degradation and ultimately valve failure. This phenomenon is closely related to material degradation, including processes such as oxidation, material fatigue, and the formation of microcracks, which promote the deposition of calcium deposits. It has been shown that calcification occurs preferentially in regions of structural damage, indicating a strong relationship between mechanical degradation and material mineralization. Consequently, this leads to further deterioration of the valve’s mechanical properties and eventual failure. These phenomena significantly limit the long-term applicability of polyurethane heart valves in blood-contacting devices and remain a major challenge in the design of durable solutions in this field [47,48].

It should be noted, however, that the susceptibility to calcification reported in classical studies predominantly concerned earlier generations of polyurethanes, particularly polyether-based formulations, in which mineralization preferentially developed at sites of fatigue-induced damage and microcrack formation [48].

Current research is focused on three complementary strategies aimed at mitigating this phenomenon. The first approach involves modification of the bulk polymer chemistry through the development of more biostable polycarbonate-based polyurethanes and systems incorporating siloxane segments or nanofillers, including PCU (polycarbonate urethane), POSS-PCU (polyhedral oligomeric silsesquioxane–polycarbonate urethane), SiPUU (siloxane poly(urethane-urea)), and FGO-PCU (functionalized graphene oxide–polycarbonate urethane). In preclinical studies, these materials demonstrated reduced susceptibility to mineralization together with improved resistance to oxidative and hydrolytic degradation [49,50,51]. An example of a contemporary polymeric valve solution is the Foldax Tria valve manufactured from the proprietary LifePolymer material, which showed no evidence of leaflet calcification in a six-month ovine preclinical model [51]. According to manufacturer-reported data, LifePolymer is characterized by high biocompatibility and biostability, favorable mechanical durability, low susceptibility to creep, and resistance to calcification [52].

The second strategy involves the development of surface modifications designed to reduce protein adsorption and platelet activation. These approaches range from classical functionalization using heparin, taurine, or aminosilanes to more advanced hydrophilic, zwitterionic, and biomimetic coatings intended to improve hemocompatibility and promote endothelialization. It has also been demonstrated that appropriately selected surface modifications may improve the fatigue resistance of polyurethane valves, whereas modifications involving polyethylene oxide (PEO) were associated with reduced durability and increased calcification [50].

The third approach focuses on optimization of polymer network architecture and manufacturing technology. Control of microphase separation, intermolecular interactions, and processing conditions may reduce creep behavior, limit microcrack initiation, and decrease secondary nucleation of calcium deposits [49].

Consequently, current research is not directed toward abandoning polyurethane-based valves, but rather toward the integrated optimization of polymer chemistry, surface properties, and microstructure in order to improve both long-term mechanical durability and resistance to calcification [49].

## 4. Material-Related Mechanisms of Thrombus Formation in MCS

The limitations of valve materials and geometry are directly connected with the broader problem of thrombogenicity in MCS. In pulsatile systems, valves, membranes, cannulae, and blood chambers form a continuous blood-contacting pathway in which surface properties and local flow conditions determine the biological response. Regions of incomplete washout, recirculation, or increased shear may promote protein adsorption, platelet activation, and thrombus formation. Therefore, thrombotic complications should not be considered separately from material selection or component design, but rather as a direct consequence of the combined effects of blood-contacting surfaces and device-related flow conditions. The analysis of thrombogenic mechanisms in MCS systems is essential for understanding the biocompatibility challenges associated with contemporary cardiac support devices [53,54,55]. Enhancements in biocompatibility through the application of advanced materials, coupled with reductions in the physical dimensions and structural complexity of MCS devices, have resulted in a significant decline in device-related complications [45,56,57]. Despite these advancements, patients continue to exhibit high morbidity rates, numerous adverse effects, and mortality [57,58,59,60,61]. These devices are characterized by sophisticated architectures comprising multiple components susceptible to mechanical failure [62]. Currently, MCS systems remain relatively invasive, technically demanding to implant, and necessitate ongoing systemic anticoagulation. Furthermore, the patient population is becoming increasingly complex, with MCS implantation being performed in progressively critical clinical states, which translates into a high incidence of perioperative complications and adverse events directly attributable to mechanical support. According to the literature data, in the early period-i.e., from insertion up to 1 month—the majority of complications associated with ECMO dominate (including bleeding, infections, thrombosis, valvular regurgitation, and mechanical failures). At the same time, for the VAD system, only bleeding, short-term inadequate support, and right heart failure are characteristic. In contrast, during the late and long-term period (beyond 1–2 weeks), ECMO complications completely subside, whereas for VAD patients, this becomes a critical phase where the risks of long-term infections, thrombosis, intracranial hemorrhage, valvular regurgitation, and mechanical failures emerge and increase [62].

Due to the limited availability of donor organs, heart transplantations are performed only for a minority of patients with advanced heart failure. Consequently, increasing attention is being directed toward the application of VAD as Destination Therapy (DT), which is well-tolerated, offers a favorable quality of life, and provides symptomatic control with a low risk of complications. Unfortunately, this risk should not be marginalized, as according to the Kaplan–Meier survival curve, it increases long-term with the duration of support. As reported by the INTERMACS (Interagency Registry for Mechanically Assisted Circulatory Support) data for continuous-flow LVAD/BiVAD implants, the overall patient survival rate systematically decreases from 95% at 1 month, to 80% at 1 year, and down to 48% at 48 months post-implant. This trend directly correlates with a late-phase rise in the hazard function (deaths per month) after the first year of support, indicating that the long-term risk of mortality and severe complications significantly intensifies over time [63].

Design improvements, successive advancements in VAD technology, and clinical experience have enabled a reduction in the incidence of the most significant complications compared to Pulsatile Flow Ventricular Assist Devices (PF-VAD). However, new complications associated with Continuous Flow Ventricular Assist Devices (CF-VAD) have emerged, including gastrointestinal bleeding related to angiodysplasia and aortic insufficiency, likely resulting from non-physiological, non-pulsatile flow and differences in left ventricular afterload [63,64].

Regardless of the nature of the blood flow through the pump-whether pulsatile or continuous-pump embolization remains one of the most critical complications during mechanical support. This phenomenon involves the entry or formation of biological embolic material within the device as a result of coagulation cascade activation. Thrombosis and thromboembolic disease are significant complications during MCS. Thrombi may develop in any part of the pump on surfaces in direct contact with blood. Bleeding and thrombotic complications are closely interrelated, and achieving an optimal balance in their prevention is often difficult. While the administration of anticoagulants and antiplatelet agents aims to mitigate the risk of these complications, no universal procedure exists for all supported patients. Elderly patients face a high risk of adverse events related to both hemorrhage and embolization, the latter of which can ultimately lead to death. Once a thrombus detaches from the pump components, it enters the bloodstream where, depending on its size and destination, it may cause various adverse effects. Neurological complications represent one of the most severe side effects resulting from MCS [65].

Virchow’s triad describes a set of three factors predisposing to thromboembolic complications [66]. These key factors include vascular wall abnormalities (e.g., endothelial damage), blood flow disturbances, and hypercoagulability. If any of these features are sub-optimal, the subsequent flow dynamics may result in thromboembolic events, potentially leading to neurological deficits or indirect ischemic complications of internal organs. Therefore, beyond the function of the pump itself, the flow domain-governed by the internal design of the device, the inflow cannula, and the outflow graft-plays a vital role. Inappropriate blood flow dynamics, along with poor positioning and fit of the cannulae, may result in circulatory stasis or generate turbulence, exposing the blood to excessive shear stress, which can initiate thromboembolic processes.

Although ventricular assist devices (VADs) are designed to minimize areas of blood stagnation that could trigger thrombus formation, this remains one of the most common complications. A key challenge lies in the fact that the thrombogenic potential of the system is a direct result of the correlation between high shear stresses and the extended residence time of activated blood components within stagnation zones. According to the model presented by Fraser et al. [67], blood flowing through the narrow channels of the device becomes susceptible to damage once the scalar shear stress (SSL) exceeds > 9 Pa. This threshold induces the mechanical degradation of von Willebrand factor (vWF) through the uncoiling of its high-molecular-weight multimers, making them vulnerable to proteolytic cleavage by the ADAMTS13 enzyme. This leads to a secondary vWF deficiency and impaired hemostasis, which paradoxically increases the risk of bleeding while maintaining a tendency toward thrombosis. A further increase in stress above > 50 Pa leads to direct platelet activation, initiating aggregation independently of biochemical stimuli. When these activated cells enter regions of low flow velocity and high residence time, they accumulate, potentially leading to thrombus crystallization within the device’s geometric niches. Under extreme conditions, where shear stresses exceed > 150 Pa, mechanical forces surpass the structural strength of the erythrocyte cytoskeleton, resulting in lethal hemolysis and the release of free hemoglobin into the plasma. These phenomena, amplified by blood stagnation in stagnant zones, create a highly thrombogenic environment where pathological rheological changes overlap with structural vessel damage. Consequently, the final clinical picture is a result of the synergy between the mechanical irritation of blood components and the iatrogenic disruption of tissue continuity. Undoubtedly, endothelial damage is an inherent element of cannulation; however, the choice of surgical technique and optimal cannula design allow for a significant reduction in peri-implant tissue trauma. Additionally, abnormal coagulation, resulting from hepatic dysfunction and the use of anticoagulant/antiplatelet medications, is frequently observed preoperatively [68]. Consequently, patients receiving a VAD are exposed to disturbances in all three elements of the aforementioned triad. Thus, it is imperative to limit or eliminate the impact of vascular wall abnormalities, flow disturbances, and coagulopathic states during the implementation of mechanical circulatory support [67].

A pivotal challenge remains the fact that the mere introduction of a foreign surface into the circulatory system triggers a cascade of physiological processes that cannot be fully controlled by surgical technique alone. Thrombus formation is one of the most frequent postoperative complications in patients utilizing blood-contacting medical devices [67,68]. Foreign surfaces implanted within the human body lack natural antithrombotic mechanisms. Instead, they activate a series of interconnected pathways leading to coagulation, which include protein adsorption, platelet adhesion and activation, thrombin generation, and complement activation [69]. These processes, initiated directly at the material–blood interface, can result in the activation of the coagulation system and, ultimately, pump embolization-compromising the therapeutic outcome and posing a direct threat to the patient’s life.

As previously noted, the occurrence of thromboembolic events associated with the use of medical devices is frequently linked to increased patient mortality. Beside sub-optimal flow dynamics and high shear stresses resulting from the device design, a pivotal cause of thrombosis is the material biocompatibility of the surface. The direct interaction between synthetic materials and circulating blood serves as a potent thrombogenic stimulus, initiating reactions at the solid–blood interface. The overall incidence of thrombosis in VAD flow pumps is estimated to be between 2% and 30%. Crucially, the risk of pump thrombosis is highest during the initial days to months following VAD implantation. The first 72 h are particularly critical, as anticoagulant prophylaxis is limited during this window due to the risk of postoperative hemorrhage. One potential solution is the optimization of blood-contacting materials through advanced surface engineering techniques to ensure a high safety profile, including hemocompatibility. Countering thromboembolic complications requires aggressive pharmacological treatment, including the administration of anticoagulants (e.g., warfarin, acenocoumarol) and platelet aggregation inhibitors (e.g., acetylsalicylic acid). The necessity for lifelong supplementation of these agents stems from the continuous exposure of blood to the artificial surfaces of the pump, which generates a persistent risk of embolic material formation [70]. In patients utilizing pulsatile flow ventricular assist devices (PF-VAD), thrombosis refers to a thrombus occurring on any blood-contacting surface of the pump (inflow cannula, outflow cannula, valves, or the surface of the membrane and blood chamber). When this complication occurs, patients are exposed to the risk of embolic stroke or device failure and face reduced survival rates [71]. A potential solution to these complications involves the development of surface coating modifications that are either passive toward thrombin and blood coagulation or capable of releasing anticoagulants in a controlled manner at a specific site rather than systemically [72]. Enhanced anticoagulation can be achieved through passive or active surface coatings. Passive coatings, such as titanium nitride (TiN) and diamond-like carbon (DLC), serve as a barrier between the material and the blood. These inorganic coatings exhibit high mechanical stability and provide corrosion protection [73]. Regarding organic coatings, three approaches to developing hemocompatible surfaces can be pursued: surface passivation to achieve minimal interaction with blood proteins and cells, immobilization of active molecules to interact with blood proteins and cells, and the promotion of endothelial cell growth [74]. The group of active coatings includes zwitterionic brush-like polymers, bioactive coatings (heparin, hirudin and its derivatives, thrombin inhibitors), and passivating proteins (albumin). Surface modifications of polyurethane involve the use of particles such as PEG and its derivatives, zwitterionic polymers, and polysaccharides [75]. The incorporation of zwitterionic starch into the polyurethane matrix allows for the formation of a stable hydration layer which, due to strong water binding, blocks protein adsorption and inhibits the activation of the coagulation cascade at its early stages [76]. Beyond these protein-repelling strategies, recent advancements have been broadened toward proactive “intelligent” surfaces that promote in situ endothelialization through nanotechnology and biomimetic cues. While zwitterionic layers focus on passive defense, nanostructured coatings—such as TiO_2_ nanotubes or polymeric nanofibers—actively mimic the natural extracellular matrix to physically guide endothelial cell adhesion and migration. This topographical influence is often augmented by biological functionalization, where the immobilization of RGD peptides, aptamers, or anti-CD34 antibodies serves as a molecular “capture” mechanism for circulating endothelial progenitor cells. By integrating these biomimetic anchors with the underlying hemocompatible matrix, these next-generation coatings transition the material from a merely inert barrier to a bioactive scaffold capable of rapidly regenerating a functional, living endothelial layer, thereby significantly reducing long-term risks of thrombosis and restenosis.

Despite the broad spectrum of modifications investigated in laboratory settings, the process of transferring these technologies into routine clinical practice is complex, and the number of commercially available solutions remains very limited. The most recognized and operationally proven example of utilizing the aforementioned strategy of active molecule immobilization is the CARMEDA^®^ technology.

The CARMEDA^®^ BioActive Surface (CBAS^®^) technology (Carmeda AB, Solna, Sweden), utilized in Berlin Heart EXCOR^®^ systems (Berlin Heart GmbH, Berlin, Germany), represents one of the few examples of the serial implementation of bioactive modifications in clinical pulsatile pumps. Its mechanism of action is based on the covalent immobilization of heparin molecules onto the polymer surface. This modified interface exhibits the capacity to bind antithrombin from the patient’s plasma, leading to the localized inhibition of thrombin and factor Xa activity. The coating design, which employs end-point attachment of the heparin chains, aims to preserve their mobility and catalytic activity while simultaneously preventing their rapid leaching by the circulating blood [77].

Despite the theoretical benefits derived from mimicking the antithrombogenic properties of the endothelium, this solution does not entirely eliminate the risk of complications. Although the coating limits non-specific protein adsorption and platelet adhesion, patients still require individualized anticoagulant pharmacotherapy, and the risk of pump embolization remains a significant clinical concern.

Importantly, this technology represents an exception in the medical market. In most other extracorporeal pulsatile pumps, polymer surfaces are utilized without additional modifying layers. This state of affairs indicates a clear technological gap and a lack of consensus regarding a universal standard for surface modification in MCS systems. Despite the existence of this solution, the lack of widespread adoption of coatings in other similar designs suggests that developing a universal method to reduce thrombogenicity at the material–blood interface remains an unresolved engineering challenge. This necessitates further research by the scientific community into the stability of such layers under long-term support conditions and their actual impact on reducing the need for systemic pharmacotherapy. Replacing conventional polymer substrates with advanced surface modifications in pulsatile pump technology therefore remains an open and high-priority research direction in the field of biomaterials engineering.

The need to intensify these efforts is particularly evident in the context of clinical management during the early postoperative period. Specifically, the first 72 h following device implantation play a pivotal role, as the risk of pump thrombosis is at its peak while anticoagulant prophylaxis remains drastically limited. Due to the high risk of postoperative hemorrhage, implementing full pharmacological therapy is often unfeasible during this window, which exposes the blood contacting the unmodified material to rapid activation of the coagulation cascade. Therefore, the development of novel surface engineering techniques capable of providing a biological shield during this critical time frame, independent of systemic drug administration, is among the most paramount tasks facing designers of modern cardiac assist systems.

## 5. Conclusions and Future Directions

Complications associated with MCS remain closely linked to the properties of materials used in device components and to the biological responses initiated at blood-contacting and tissue-contacting surfaces. Despite substantial advances in pump design, adverse events such as thrombosis, embolization, local infection, inflammation, and bleeding remain strongly influenced by processes occurring at these interfaces. Protein adsorption, platelet adhesion and activation, initiation of the coagulation cascade, complement activation, and microbial adhesion are not independent phenomena, but interconnected responses triggered by contact between blood, tissue, and artificial materials.

This review focused particularly on first-generation pulsatile systems, which remain clinically important in pediatric MCS. The use of extracorporeal pumps, permanent cannula exit sites, polymeric blood chambers, membranes, and adapted valve components creates multiple locations where material properties, surface characteristics, and local flow conditions directly influence clinical outcomes. In these systems, complications related to materials are especially relevant because the device must simultaneously maintain hemocompatibility, resist infection, tolerate cyclic mechanical loading, and preserve stable flow through the pump.

The complications discussed in this review were selected because of their direct association with materials in contact with blood or tissue. Persistent exit-site infections reflect the difficulty of maintaining stable tissue integration around percutaneous components exposed to mechanical stress and microbial contamination. Thrombus formation within the pump reflects the thrombogenic potential of artificial blood-contacting surfaces, particularly under conditions of disturbed flow, incomplete washout, or elevated shear stress. Valve-related limitations further illustrate the same problem: components adapted from intracardiac applications may not be optimal for extracorporeal pump environments, where geometry, material selection, durability, and hemocompatibility must be considered together.

Current research is therefore directed toward a more integrated approach to material design. Strategies aimed at reducing thrombogenicity include bioactive and biomimetic coatings capable of limiting protein adsorption, platelet adhesion, and thrombin generation at the material surface. These approaches are particularly relevant in the early postoperative period, when systemic anticoagulation is often limited by bleeding risk. In parallel, infection prevention requires materials and surface modifications that reduce bacterial adhesion, delay biofilm maturation, and maintain acceptable tissue compatibility during long-term support. This is especially important for percutaneous components, which remain exposed to repeated handling, local mechanical stress, and the hospital environment.

Future development of MCS systems should not rely on isolated improvements in a single device component. Progress will require coordinated optimization of material chemistry, surface topography, coating stability, mechanical properties, and device geometry. Dedicated valve designs for pulsatile pumps, improved antibacterial and hemocompatible surfaces, and more durable polymeric materials represent complementary directions rather than separate solutions. In this context, the reduction in complication rates will depend not only on advances in pump technology, but also on the ability to control biological responses at material interfaces. This approach is particularly relevant for pediatric MCS, where anatomical constraints, prolonged support, and narrow therapeutic margins amplify the clinical consequences of material-related complications.

## Data Availability

No new data were created or analyzed in this study. Data sharing is not applicable to this article.
